# Non-Metastatic Clear Cell Renal Cell Carcinoma Immune Cell Infiltration Heterogeneity and Prognostic Ability in Patients Following Surgery

**DOI:** 10.3390/cancers16030478

**Published:** 2024-01-23

**Authors:** Daniel D. Shapiro, Taja Lozar, Lingxin Cheng, Elliot Xie, Israa Laklouk, Moon Hee Lee, Wei Huang, David F. Jarrard, Glenn O. Allen, Rong Hu, Toshi Kinoshita, Karla Esbona, Paul F. Lambert, Christian M. Capitini, Christina Kendziorski, Edwin Jason Abel

**Affiliations:** 1 Department of Urology, University of Wisconsin School of Medicine and Public Health, Madison, WI 53792, USA; 2 William S. Middleton Memorial Veterans Hospital, Madison, WI 53705, USA; 3 McArdle Laboratory for Cancer Research, University of Wisconsin, Madison, WI 53706, USA; 4 Department of Biostatistics and Medical Informatics, University of Wisconsin School of Medicine and Public Health, Madison, WI 53792, USA; lcheng74@wisc.edu (L.C.);; 5 Department of Pathology, University of California, Los Angeles, Los Angeles, CA 90024, USA; ilaklouk@mednet.ucla.edu; 6 Department of Pathology, University of Wisconsin School of Medicine and Public Health, Madison, WI 53792, USArhu6@wisc.edu (R.H.); kesbona@wisc.edu (K.E.); 7 Department of Pediatrics, University of Wisconsin School of Medicine and Public Health, Madison, WI 53792, USA; ccapitini@pediatrics.wisc.edu

**Keywords:** renal cell carcinoma, tumor heterogeneity, immune microenvironment, T cell

## Abstract

**Simple Summary:**

It is difficult to predict which patients with non-metastatic clear cell renal cell carcinoma (ccRCC) will develop metastatic disease after nephrectomy. Recent studies suggest that immune cell infiltration within ccRCC tumors may impact tumor progression. This study assessed the number and type of immune cells in non-metastatic ccRCC tumors that were surgically removed and their ability to predict which patients developed metastatic disease. We found that higher levels of a specific immune cell (CD8^+^ T cells) were linked to a lower risk of progressive disease. Patients who did progress had more exhausted CD8^+^ T cells in the tumor microenvironment. Additionally, our study design accounted for tumor heterogeneity by sampling tumors in multiple locations and showed differences in the spatial distribution of CD8^+^ T cells in tumors that progressed to metastatic disease. With further validation, this study shows that CD8^+^ T cell infiltration within ccRCC tumors could be used as a prognostic biomarker to predict progression to metastatic disease.

**Abstract:**

Predicting which patients will progress to metastatic disease after surgery for non-metastatic clear cell renal cell carcinoma (ccRCC) is difficult; however, recent data suggest that tumor immune cell infiltration could be used as a biomarker. We evaluated the quantity and type of immune cells infiltrating ccRCC tumors for associations with metastatic progression following attempted curative surgery. We quantified immune cell densities in the tumor microenvironment and validated our findings in two independent patient cohorts with multi-region sampling to investigate the impact of heterogeneity on prognostic accuracy. For non-metastatic ccRCC, increased CD8^+^ T cell infiltration was associated with a reduced likelihood of progression to metastatic disease. Interestingly, patients who progressed to metastatic disease also had increased percentages of exhausted CD8^+^ T cells. Finally, we evaluated the spatial heterogeneity of the immune infiltration and demonstrated that patients without metastatic progression had CD8^+^ T cells in closer proximity to ccRCC cells. These data strengthen the evidence for CD8^+^ T cell infiltration as a prognostic biomarker in non-metastatic ccRCC and demonstrate that multi-region sampling may be necessary to fully characterize immune infiltration within heterogeneous tumors. Tumor CD8^+^ T cell infiltration should be investigated as a biomarker in adjuvant systemic therapy clinical trials for high-risk non-metastatic RCC.

## 1. Introduction

Approximately two-thirds of renal cell cancer (RCC) patients have clinically localized disease at presentation, for which surgery is commonly recommended [1]. Metastatic progression is exceptionally uncommon for stage 1 tumors (<5%) following surgery. However, the risk of metastatic progression increases with higher stage tumors and varies significantly among patients. For example, in patients with pT2 (tumors of ≥7 cm confined to the kidney), ~30% of patients progress from non-metastatic to metastatic disease, and pT3 tumors (non-metastatic tumors invading the perinephric fat or venous system) have a progression rate of ~50–70% [2,3,4,5]. Current prognostic models use clinical and pathological variables but have limited prognostic ability to identify patients who are at high risk of developing metastatic disease after surgery [6]. Correa et al. demonstrated that commonly used prognostic models perform poorly, with limited predictive accuracy demonstrated by c-indices ranging between 0.56 and 0.69 [6].

Currently, biomarkers for RCC are not used clinically for localized, locally advanced, or metastatic RCCs. However, biomarkers are critically needed to guide treatment decisions for patients with all stages of disease. Useful biomarkers must be developed and validated using independent cohorts, and they should provide actionable information that informs decision-making. As such, high-risk non-metastatic RCC patients are an ideal cohort for biomarker-informed decision-making because patients have to choose whether or not to be treated with adjuvant immune checkpoint inhibitor therapy after surgery [7,8]. Potential benefits of treatment include improved disease-free survival compared to placebo. However, patients treated with adjuvant therapy also have a 32% overall risk of adverse events and a 20% risk of serious adverse events [8]. In this subpopulation, prognostic biomarkers to improve prognostic ability may facilitate treatment of the patients most likely to develop metastatic disease while avoiding adverse events in lower risk patients.

There is a strong biologic rationale to investigate the immune microenvironment as a prognostic biomarker for non-metastatic clear cell RCC because it is one of the most highly immune-infiltrated solid tumors [9,10]. Additionally, metastatic ccRCC is highly responsive to treatment with immune checkpoint inhibitor therapy (ICI), with multiple approved ICI therapies for metastatic disease and adjuvant therapy [8,11,12,13,14,15]. To date, a few studies have investigated the immune microenvironment of non-metastatic ccRCC as a prognostic biomarker, and studies that have investigated the immune microenvironment have demonstrated conflicting results regarding the prognostic capability of infiltrating immune cells [16,17,18]. The objective of this study was to evaluate the prognostic value of immune infiltration within non-metastatic ccRCC following surgery and investigate whether immune cell heterogeneity, cellular composition, and spatial distribution of immune cells are correlated with prognosis.

## 2. Materials and Methods

### 2.1. Discovery Cohort Patient Selection

**Figure 1:**
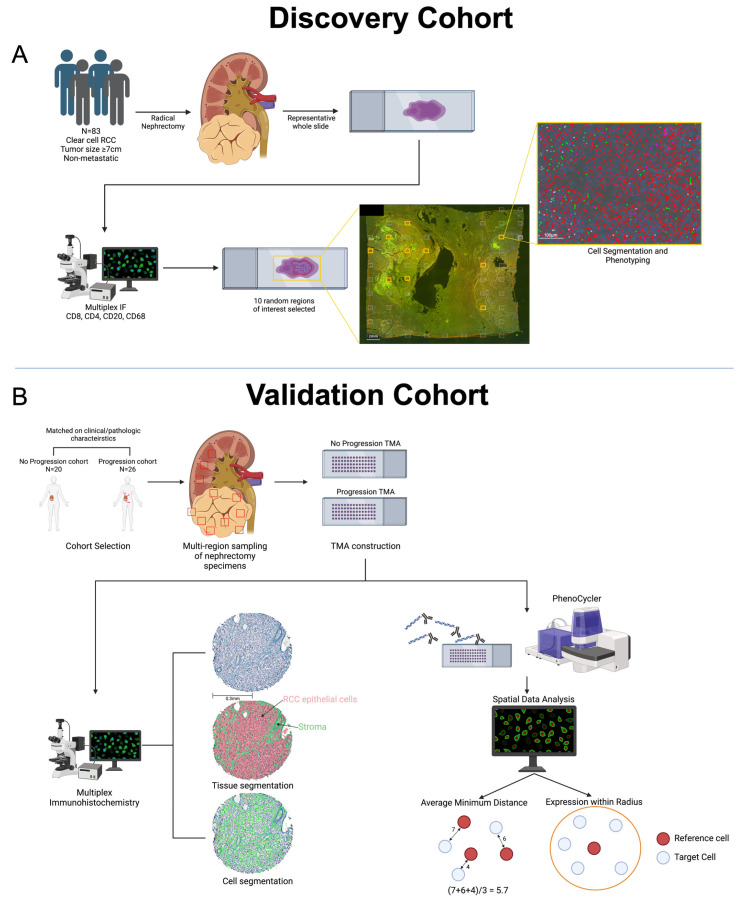
Evaluation of the immune microenvironment for surgically resected non-metastatic clear cell renal cell carcinoma. Two cohorts of patients were used to evaluate the immune microenvironment. (**A**) A discovery cohort consisted of 83 patients with non-metastatic ccRCC with tumors ≥7 cm who underwent radical nephrectomy. Representative whole slides were selected for each case containing the greatest degree of tumor-infiltrating lymphocytes. These slides underwent multiplex immunofluorescence for immune cell markers. Ten regions of interest were selected randomly from each slide, and these regions underwent further cell segmentation, cell phenotyping, and subsequent quantification. (**B**) Findings from the discovery cohort were validated in a separate cohort of 46 patients with large (≥7 cm) non-metastatic ccRCC tumors. The patients used for the validation cohort were split into two groups (progression to metastatic disease versus no progression to metastatic disease), and these groups were matched on clinical characteristics that have previously been demonstrated to be prognostic for metastatic progression. Tissue microarrays (TMAs) were constructed from nephrectomy specimens. Each tumor underwent multi-region sampling, including 7–8 different tumor regions and 3 samples of non-adjacent normal renal parenchyma. These 10–11 cores from each tumor were used to construct the TMAs. The tissue microarrays were stained using multiplex immunohistochemistry for markers of immune cells and then quantified. The TMAs were additionally stained using the PhenoCycler multiplex immunofluorescence platform for further spatial quantification and measurements of immune cell exhaustion.

Institutional review board approval at the University of Wisconsin—Madison was obtained for this study (#2018-0018). Patients from a single center were included if they underwent surgery for non-metastatic ccRCC. Patients were eligible if they had a primary tumor with a maximum pathologic tumor diameter ≥7 cm in size (AJCC T stage 2) with no clinical evidence of nodal or other metastatic disease (Figure 1A). Clinical and pathologic data were extracted from medical records. All pathologic slides from each tumor were evaluated by a trained genitourinary pathologist. A representative whole slide was selected that contained tumor tissue and the highest degree of lymphocyte infiltration for further quantitative analysis.

### 2.2. Discovery Cohort Histology and Immunostaining

After the selection of a representative slide from each tumor, additional slides were cut from archival FFPE tissue blocks. Slides were sectioned at 5 μm thickness and mounted on positively charged glass slides. Multiplex immunostaining was performed using the Opal method following the manufacturer’s protocol (Akoya, Boston, MA, USA), and antibodies were applied in sequence as follows: CD4/Opal 540 (Ventana 790-4423, RTU, 10 min), CD68/Opal 570 (Ventana 790-2931, RTU, 5 min), CD8/Opal 620 (Ventana 790-4460, RTU, 30 min), CD20/Opal 650 (Ventana 790-4431, RTU, 15 min), and Pan-Cytokeratin (PCK)/Opal 570 (Abcam, ab234297, 1:1500, 15 min).

### 2.3. Discovery Cohort Slide Image Acquisition and Analysis

After staining, tissue section slides were scanned with Vectra 2 (Akoya, Boston, MA, USA) at 4× magnification, and then 10 fields of view were randomly selected per slide with PhenoChart software (Akoya, Boston, MA, USA) to be scanned with Vectra 2 at 20× magnification and analyzed with the InForm v.2.4 software (Akoya, Boston, MA, USA). A spectral library algorithm was created to unmix each individual signal, and the following pseudocolors were applied for image analysis: CD4 (yellow), CD68 (red), CD8 (green), CD20 (pink), and PCK (magenta). The InForm software was used to segment tissue compartments (epithelium vs. stroma) and subcellular compartments (nucleus, membrane, and cytoplasm). Individual cell segmentation was performed, and cell phenotypes were quantitated as cell density (cells/mm^2^) (Figure 1A).

### 2.4. Validation Tissue Microarray Construction

To validate the findings of the discovery cohort as well as account for tissue heterogeneity, a validation cohort tissue microarray (TMA) was constructed. Tumors included in the TMA included two cohorts of patients who underwent surgery for non-metastatic ccRCC: those who progressed to metastatic disease (N = 26) and those who did not progress at last follow-up (N = 20) (Figure 1B). These patients were separate from the patients included in the discovery cohort. In order to account for potential other factors that may influence progression beyond the immune microenvironment, the patients in the progression and no progression cohorts were matched on age at the time of surgery, gender, performance status, preoperative serum C-reactive protein (CRP), neutrophil-lymphocyte ratio (NLR), pathologic stage, tumor size, tumor grade, and the presence of tumor thrombus. In order to account for differences in immune infiltration spatially throughout the tumor (i.e., spatial heterogeneity), the TMA was constructed to contain at least 7–8 tumor sample cores from each tumor, which were spatially distributed throughout the tumor. Additionally, 3 non-adjacent, non-tumor tissue cores of normal renal parenchyma were included for comparison. The TMA was constructed as previously described using a Manual Tissue Array (Beecher Instruments, Sun Prairie, WI, USA; model MTA-1), with 0.6 mm cores arranged 0.8 mm apart [19].

### 2.5. Validation of TMA-Multiplexed Immunohistochemistry

For this, 4 μm thick TMA slides were deparaffinized, and heat-induced epitope retrieval was carried out. Each slide underwent triple staining with two immune markers of interest and smooth muscle actin (SMA). For example, the progression and no progression TMA slides were incubated with the first primary antibody (CD4 Ventana #790-4423), then the slide was rinsed, and the Discovery OmniMap anti-Rabbit HRP (Ventana #760-4311) was applied. Slides were rinsed, and Reaction Buffer (Ventana #950-300) and Discovery ChromoMap DAB detection were applied (Ventana #760-159). Discovery Inhibitor (Ventana #760-4840) was applied, and then the slide was incubated with the second SMA antibody (Ventana #760-2833). Discovery OmniMap anti-mouse HQ (Ventana #740-4814) was applied with anti-HQ-HRP (Ventana #760-4820). The slide was rinsed, the Discovery Teal HRP detection kit (Ventana #760-247) was applied, and the denaturing agent Discovery Inhibitor (Ventana #760-4840) was applied. The slide was incubated with the primary anti-CD8 antibody (Ventana #790-4460), rinsed, and the Discovery UltraMap anti-rabbit AP (Ventana #760-4314) was applied. The slide was rinsed, and the Discovery Red HRP detection kit (Ventana #760-228) was applied. The slide was then washed, counterstained with hematoxylin, rinsed, dehydrated, and dipped in xylene. A similar protocol was used for our two additional markers of interest: CD20 (Ventana #760-2531) and CD68 (Ventana #790-2931).

### 2.6. Tissue Microarray Automated Image Acquisition and Analysis

Tissue microarray automated image acquisition and analysis followed a similar protocol as previously described [19]. Stained slides were loaded into the Vectra 2 slide scanner (Akoya, Boston, MA, USA), and an automated scanning protocol was created to acquire multi-spectral image cubes using the 20× objective. Control slides stained with only 1 chromogen were used to create a spectral library in Nuance v3.0.2 software (Akoya, Boston, MA, USA). Image cubes were opened in InForm v2.4 software, and images were chosen to set up an algorithm of differentiation for tissue and cell segmentation [19]. The algorithm was applied to the full set of TMA image cubes, and the expression of markers was quantified using segmentation settings sufficient to cover the cell membrane compartment. Protein expression data was exported, and cell density was calculated as cells/mm^2^.

### 2.7. Tissue Microarray Staining and Image Acquisition Using the PhenoCycler Platform

We additionally performed high multiplex immunofluorescence using the PhenoCycler platform in order to understand in greater detail the spatial arrangement of cells within the tumor microenvironment using the validation TMAs [20]. Formalin-fixed paraffin-embedded validation TMA sections were analyzed using the PhenoCyclerTM-Open (formerly CODEX) platform (Akoya, Boston, MA, USA). Tissue was cut at 5 µm thickness and mounted onto superadhesive slides. The FFPE TMA tissue sections were dewaxed and rehydrated following standard histology methods. Epitope retrieval was performed using Tris-EDTA pH 9 for 20 min in a programmable pressure cooker (Instant PotTM). After allowing the pressure cooker to cool and depressurize naturally, the tissue was bleached by immersion in a solution of 4.5% (*w*/*v*) H202 and 20 mM NaOH in PBS under bright white LED light (A4-sized, Aibecy A4 Ultra Bright 25,000 Lux LED Light Box-Tracing Pads). The TMA was stained with a mixture of oligonucleotide-barcoded PhenoCycler antibodies (Supplementary Table S1) and post-fixed, according to the user manual. Imaging experiments were performed with the PhenoCyclerTM connected to a Keyence BZ-X800 epifluorescence microscope with a 20× objective (Nikon CFI Plan Apo 20×/0.75) (Figure 1B). The multiplex cycles were set up using Akoya’s CODEX Instrument Manager (CIM), and the acquired images were then processed with the PhenoCyclerTM Processor to perform cycle alignment, background subtraction, deconvolution, extended depth of field, shading correction, tile registration, and stitching. The resulting QPTIFF image files were manually inspected for quality using the QuPath v0.5.0 software.

### 2.8. Cell Phenotype Labeling with PhenoCycler-Generated Images

After QPTIFF generation, QuPath v0.4 was used to process the 22-channel QPTIFF image generated from the PhenoCycler instrument. Cell segmentation is the first step of image analysis, which involves the identification of individual cells within the TMA cores and their corresponding 2-D x and y coordinates. Cell segmentation was performed with the StarDist (arXiv:1806.03535) nuclear segmentation algorithm using the DAPI channel and exported as a text file. Once cells were segmented, the CELESTA (cell-type identification with spatial information) algorithm was used to automate cell-type identification in our multiplexed image data [21]. CELESTA uses both protein expression and cell spatial neighborhood information from segmented imaging data for automated, unsupervised machine learning cell type identification. CELESTA requires two inputs, including the segmented imaging data as well as a cell-type signature matrix, which contains the cell types to be inferred from the markers used. For the purposes of this paper, we focused our matrix on Pan-Cytokeratin (PCK^+^) malignant ccRCC cells, CD45^+^CD3e^+^CD8^+^ T cells, and within CD8^+^ T cells, and we defined CD8^+^ T cells as exhausted if they co-expressed PD1^+^ with LAG3^+^ or PD1^+^ with TIM3^+^ [22,23,24]. CELESTA performs cell phenotype assignments and allows for cells to be plotted in two dimensions. Initially, quality control was conducted by excluding cells that exhibited either uniformly high or uniformly low expression across all markers, ensuring the removal of potential outliers or artifacts. Following the cell assignment outputs from the CELESTA algorithm, post-quality assessments were carried out by comparing these assignments with the original images obtained from the PhenoCycler platform. To further refine cell-type identification, we manually adjusted the ‘high_expression_threshold’ parameter for each cell type. This threshold, defining the minimum marker expression probability required for a marker to be considered as expressed, was determined by carefully comparing the expression probabilities against the corresponding PhenoCycler staining patterns for each marker.

### 2.9. Statistical Analysis and Spatial Analysis

Differences in clinical and pathologic characteristics were compared using the Wilcoxon rank-sum test and Fisher’s exact test for continuous and categorical variables, respectively. The density of cell types was compared between patients who progressed versus those who did not progress using a mixed effects model, given that multiple samples (i.e., technical replicates) were taken from individual tumors from each patient. Survival analysis was performed using the Kaplan–Meier method, and differences in survival outcomes were estimated using the logrank test. When stratifying by immune cell density and comparing survival differences, the cell densities of each immune cell were averaged at the patient level. Patients were stratified into high- and low immune cell cohorts based on the median immune cell density. A Cox proportional hazard model was used to evaluate the association between survival and immune cell density. Logistic regression was used to evaluate the association between early progression and immune cell density. We used the PhenoCycler multiplex immunofluorescence data to evaluate the association of exhausted CD8^+^ T cells. We evaluated cores with higher than the median percent of tumor cells and lower than the median CD8^+^ T cell percentage as indicative of a “poor” immune response. For spatial analyses, TMA cores were categorized based on the median CD8^+^ cell percentage within the progression and no progression cohorts. Subsequently, calculations were made to determine both the mean percentage of CD8^+^ cells surrounding malignant cells and the average minimum distance between malignant cells and CD8^+^ cells in the TMA cores with high CD8^+^ T cell infiltration. These metrics were then compared between patients who progressed and those who did not, utilizing the Wilcoxon rank-sum test. The spatial analysis was conducted using SPIAT (v.1.2.3) [25]. The coefficient of variation (CV) was calculated for each tumor according to the following equation: CV=σ/μ, where σ = the standard deviation of the cell density and μ = the mean cell density. The Wilcoxon rank-sum test was used to compare the CV between the progression versus no progression cohorts. Two-tailed *p* values < 0.05 were considered statistically significant. The statistical software used for analysis included STATA^®^ SE v18 (StataCorp, College Station, TX, USA), GraphPad Prism v10.0.2 (GraphPad Software, Boston, MA, USA), and R v4.3.0.

## 3. Results

### 3.1. Discovery Cohort to Evaluate the Prognostic Impact of CD8^+^ T Cells

First, we evaluated whole slide images of non-metastatic ccRCC cases that were surgically resected at a single institution for patients with tumors ≥7 cm. Pathologic slides were evaluated by a trained GU pathologist, and representative slides were obtained from 83 cases. The cohort was divided based on metastatic progression at the last follow-up. Clinical and pathologic characteristics are listed in Table 1. Patients in the progression group were slightly older and tended to have higher grade disease. Median follow-up was similar in both groups, with a median follow-up of 37 months for those that did not progress versus 33 months for those that did progress (*p* = 0.9).

**Figure 2:**
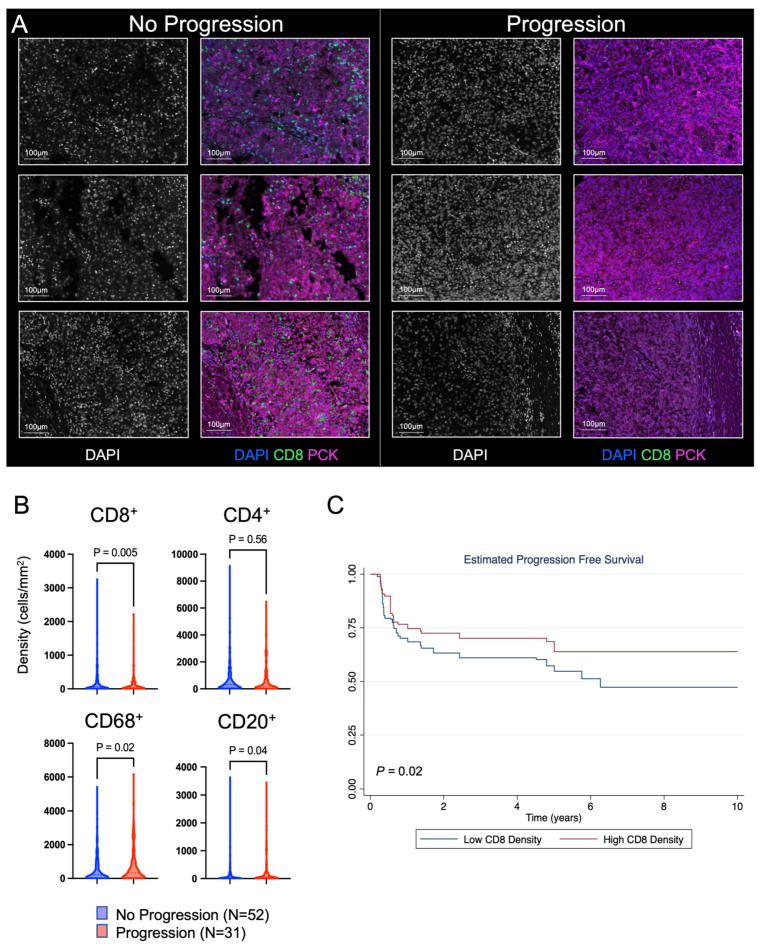
Discovery cohort immune microenvironment characteristics. (**A**) Representative multiplex immunofluorescence images of tumor tissue. The tissue images shown here were obtained by randomly selecting 3 patients in the no progression and progression cohorts. Within each cohort, the images on the left demonstrate the DAPI-stained individual cell nuclei, and the images on the right demonstrate the unmixed images, including the markers for DAPI, CD8 (effector T cell marker), and PCK (tumor cell marker). (**B**) Cell densities of individual immune cells for patients that either progressed to metastatic disease or did not. A mixed-effects model was used for comparison between the two groups. (**C**) Progression-free survival analysis. CD8^+^ T cell densities were averaged at the individual patient level, and then patients were stratified by the overall median CD8^+^ T cell density into high- and low-density cohorts. Differences in progression-free survival were calculated using the logrank test.

Representative slides from each tumor were then sampled in 10 randomly selected regions to quantify the density of individual immune cells (Figure 1A). Slides were stained using multiplex immunofluorescence for CD4^+^ T cells, CD8^+^ T cells, CD68^+^ macrophages, and CD20^+^ B cells (Figure 2A). Based on other solid tumor types, we hypothesized that the CD8^+^ T cell density would be higher among patients that did not progress. We indeed saw a higher mean CD8^+^ T cell density among patients that did not progress (196.2 vs. 129.1 cells/mm^2^, *p* = 0.005). We additionally found a higher density of CD68^+^ macrophages and CD20^+^ B cells among patients that progressed (Figure 2B). We then evaluated progression-free survival among patients in the discovery cohort. We calculated the average CD8^+^ T cell density from each patient’s tumor and stratified patients by the median CD8^+^ T cell count from the entire discovery cohort. Patients stratified by high CD8^+^ T cell density had improved progression-free survival (logrank *p* = 0.02) and a reduced risk of metastatic progression after surgery for localized ccRCC (HR 0.67, 95% CI 0.47–0.96; *p* = 0.03) (Figure 2C).

### 3.2. Validation Cohort to Evaluate the Prognostic Impact of CD8^+^ T Cells

In order to validate the findings from the discovery cohort and reduce the risk of confounding factors that could influence prognosis and the degree of immune infiltration within the tumor microenvironment, we constructed TMAs of patients who either progressed or did not progress to metastatic disease after surgery for localized ccRCC. These TMAs consisted of patients with tumors ≥7 cm, and patients were matched on age at the time of surgery, gender, performance status, preoperative serum CRP, NLR, pathologic stage, tumor size, grade, and the presence of tumor thrombus. Multi-region sampling was performed, and tumors were sampled in seven to eight separate locations throughout the renal mass. Median follow-up for progressed and not progressed cohorts was 7 and 11 years from the date of surgery (Figure 1B). Table 2 demonstrates the validation cohorts’ clinical and pathological characteristics. The progression and no progression cohorts had no significant differences between any of the matching criteria.

**Figure 3:**
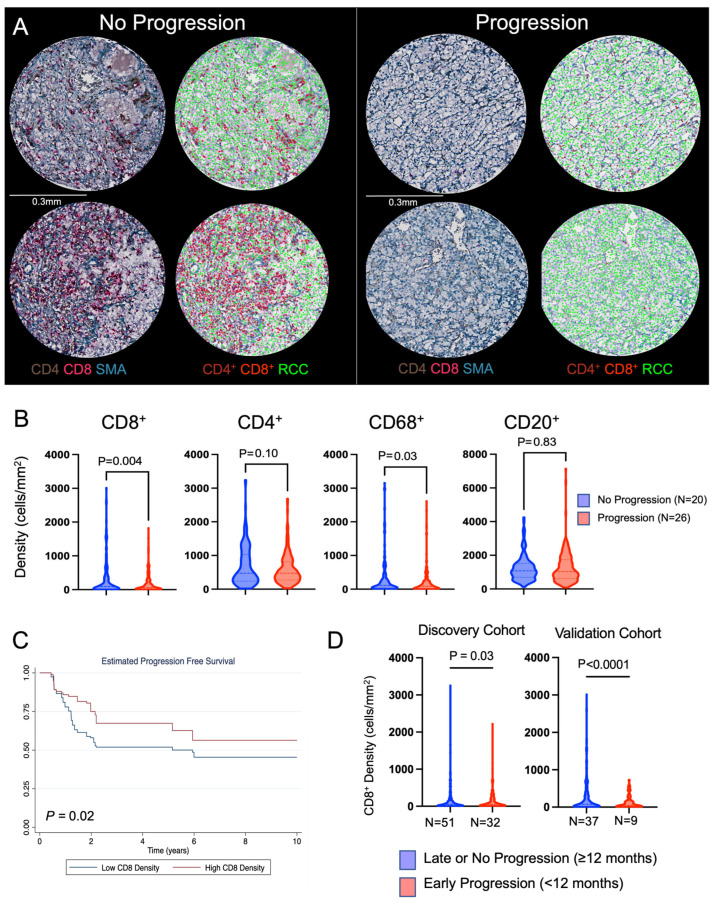
Validation cohort immune microenvironment characteristics. (**A**) Representative multiplex immunohistochemistry (IHC) images of tumor tissue. The tissue cores shown here were obtained by randomly selecting 2 patients from the no progression and progression cohorts. Within each cohort, the images on the left demonstrate brightfield IHC staining for CD4, CD8, and SMA (smooth muscle actin). The images on the right are the corresponding cell phenotypes as defined by the automated cell segmentation algorithm, with cell types being CD4^+^ T cells in brown, CD8^+^ T cells in red, and ccRCC cells in green. (**B**) Cell densities of individual immune cells for patients that either progressed to metastatic disease or did not. A mixed-effects model was used for comparison between the two groups. (**C**) CD8^+^ T cell densities were averaged at the individual patient level, and then patients were stratified by the overall median CD8^+^ T cell density into high- and low-density cohorts. Differences in progression-free survival were calculated using the logrank test. (**D**) For both the discovery and validation cohorts, patients were separated into those that progressed within 12 months of surgery and those that progressed after 12 months of surgery or did not progress at the last follow-up. A mixed-effects model was used to compare CD8^+^ T cell densities between early and late/no progression groups.

Immunohistochemistry staining was performed for markers of immune cells and quantified. As with the discovery cohort, the validation TMAs were stained for CD4^+^ T cells, CD8^+^ T cells, CD68^+^ macrophages, and CD20^+^ B cells (Figure 3A). Similar to the discovery cohort, an increased CD8^+^ T cell density was demonstrated within the tumor immune microenvironment of localized ccRCC tumors that did not progress compared to those that did progress (median 330.6 cells/mm^2^ vs. 181.5 cells/mm^2^, *p* = 0.004). We did not find similar patterns among other immune cell markers that were found within the discovery cohort (Figure 3B). As with the discovery cohort, the CD8^+^ T cell density was then averaged per patient, and patients were stratified by the median CD8^+^ T cell density to create low- and high-density groups. Survival analysis again demonstrated that tumors highly infiltrated by CD8^+^ T cells had a better prognosis (Figure 3C) and had a reduced risk of progression compared to tumors that had lower CD8^+^ T cell infiltration (logrank *p* = 0.02).

We noted that a subset of patients in both the discovery (32/83, 39%) and validation (9/46, 20%) cohorts progressed early (<12 months after surgery). In our discovery cohort, we found that the mean CD8^+^ T cell density was lower in patients that progressed early (<12 months) after surgery compared to patients that progressed ≥12 months after surgery or did not progress (137.2 cells/mm^2^ vs. 189.5 cells/mm^2^, *p* = 0.03). Patients with higher CD8^+^ T cell density had reduced odds of early recurrence (OR 0.99, 95% CI 0.98–0.99; *p* = 0.04). The validation cohort had similar findings, with a mean CD8^+^ T cell density of 128.9 cells/mm^2^ in patients that progressed early compared to 275.1 cells/mm^2^ among patients that progressed ≥12 months after surgery or did not progress (OR 0.99, 95% CI 0.98–0.99; *p* = 0.02) (Figure 3D).

**Figure 4:**
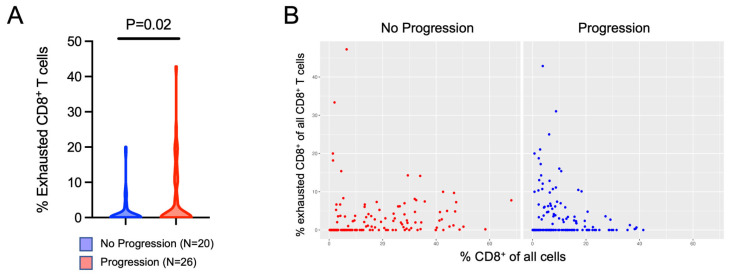
Evaluation of T cell exhaustion. Cell phenotyping was performed using the PhenoCycler multiplex immunofluorescence data. (**A**) The percentage of exhausted CD8^+^PD1^+^LAG3^+^ T cells from all CD8^+^ T cells was compared between patients that progressed versus those that did not progress. A mixed-effects model was used to compare the two cohorts. (**B**) The percentage of CD8^+^ T cells was calculated from all cells present in individual tumor cores (x-axis). This was compared to the percentage of exhausted (CD8^+^PD1^+^LAG3^+^) T cells from the total CD8^+^ T cell population (y-axis).

We hypothesized that the specific CD8^+^ T cell phenotype may be associated with progression to metastatic disease and that a higher percentage of exhausted CD8^+^ T cells would be associated with a higher risk of progression. Using the PhenoCycler platform, we evaluated markers of T cell exhaustion. We found that a higher percentage of exhausted (CD8^+^PD1^+^LAG3^+^) T cells were present in patients that recurred (Figure 4A). Additionally, we found that among patients who did not progress, the percent of exhausted CD8^+^ T cells among all CD8^+^ T cells was relatively stable regardless of how infiltrated the tumor tissue was. In the patients that progressed, however, there was a higher proportion of exhausted CD8^+^ T cells among tumor cores that had lower CD8+ T cell infiltration, suggesting that tumors that progressed were more likely to have a weak immune response indicated by a lower CD8^+^ T cell infiltration combined with a higher proportion of exhausted CD8^+^ T cells (Figure 4B). Lastly, using logistic regression, we confirmed that an increasing percent of exhausted PD1^+^LAG3^+^CD8^+^ T cells was associated with increased odds of progression to metastatic disease (OR 1.39, 95% CI 1.02–1.90, *p* = 0.038).

### 3.3. Evaluation of Tissue Heterogeneity and Spatial Variation of Immune Cell Infiltration within Non-Metastatic ccRCC

**Figure 5:**
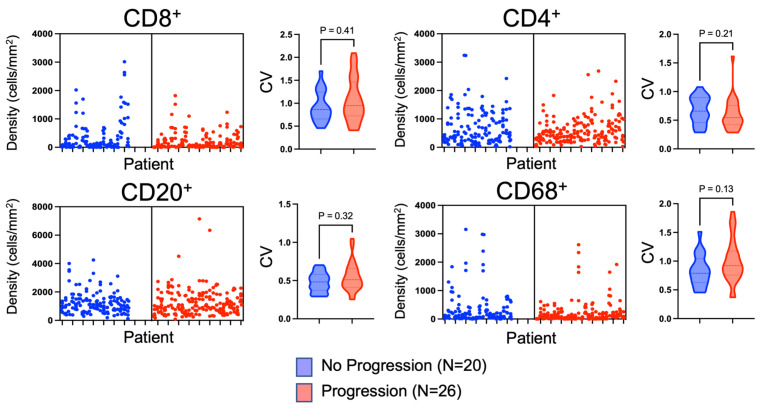
Evaluation of the heterogeneity. The cell densities of individual RCC cores were plotted for each patient, stratified by patients that progressed and those that did not progress, to visualize the variation in immune cell densities at the individual patient level. The x-axis of the cell density plots represents an individual patient. The cell density of each TMA core from that patient’s tumor is represented by an individual dot stacked vertically. To quantify immune cell density heterogeneity, coefficients of variation (CV) were calculated between patients that progressed and did not progress, as represented by the violin plots.

Given that the validation TMAs were constructed using multi-regional sampling, we were able to use these TMAs to investigate the immune cell heterogeneity throughout individual RCC tumors. Figure 5 demonstrates the immune cell density within individual cores from each patient’s ccRCC tumor. Substantial variation existed among individual cores. To quantify this variation, coefficients of variations (CVs) were calculated for each tumor, and the median CVs were compared between patients that progressed versus patients that did not progress. All immune cell markers had high CVs, with the highest being CD8^+^ and CD68^+^. CD20^+^ B cell density had the lowest CV (Figure 5 and Table 3). No difference was found among the CVs for patients that progressed versus those that did not progress, indicating that all tumors, regardless of their capacity to progress, had substantial intratumoral immune cell density heterogeneity (Figure 5 and Table 3).

**Figure 6:**
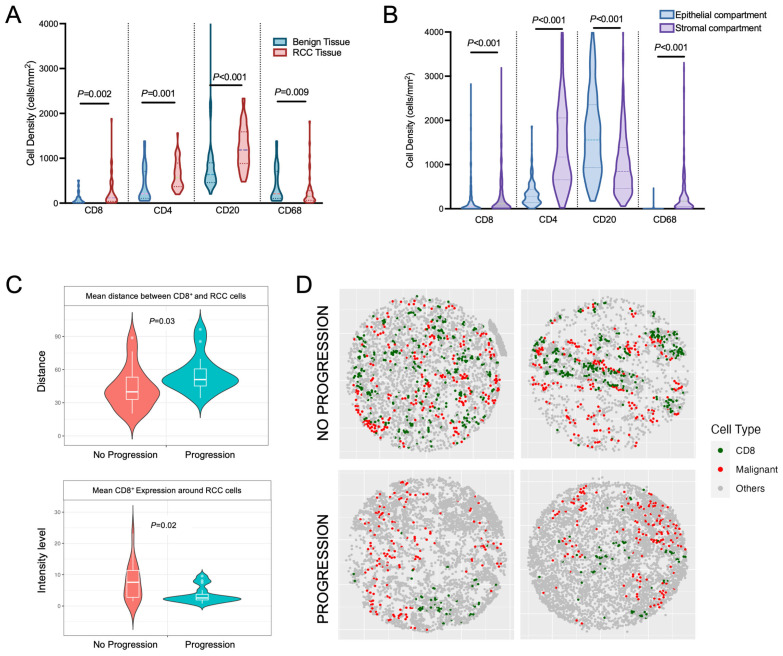
Evaluation of the spatial distribution of immune cell markers. (**A**) Patients within the validation cohort had immune cell markers quantified and compared between RCC tissue and non-adjacent normal renal parenchyma. (**B**) Using the validation cohort, tumor tissue cores were separated into stromal and epithelial compartments. The density of immune cells within each compartment was quantified and compared. Statistical comparisons of the mean cell densities between benign and RCC tissue as well as between stroma and epithelial compartments were made using the Wilcoxon paired signed rank test. (**C**) Validation TMAs were stained using the PhenoCycler platform and analyzed for spatial distribution of CD8^+^ T cells in relation to malignant RCC cells (Pan-cytokeratin positive). The mean minimum distance between CD8^+^ T cells and malignant cells was compared between the two cohorts. Also, the mean CD8^+^ expression was calculated within a predefined radius of 150 pixels and compared between the progression and no progression cohorts. (**D**) Two representative TMA cores from the no progression and progression cohorts demonstrate differences in the spatial organization of CD8^+^ T cells and ccRCC cells.

To evaluate how immune cells infiltrated the kidney and RCC tumors, we assessed differences in benign versus tumor immune cell infiltration using the validation TMA, which captured both benign renal parenchyma and corresponding tumor tissue for comparison. As expected, we found that tumor tissue contained a significantly higher degree of immune cell infiltration among all markers assessed (Figure 6A). We then evaluated immune cell penetration among the tumor epithelial cells versus within the surrounding stroma by segmenting each TMA core into epithelial and stromal compartments. Within the individual TMA RCC cores, immune cell infiltration was significantly less within the epithelial compartment, while the stromal compartment contained the majority of immune-infiltrating lymphocytes, except for CD20^+^ cells, which were more prevalent within the epithelial compartment (Figure 6B). Overall, we demonstrate that the immune infiltration within the surrounding kidney is less than the tumor tissue. Additionally, within the RCC tissue, the majority of the immune infiltration occurs within the stromal tissue surrounding the tumor epithelial cells.

Using the PhenoCycler analysis of the validation TMAs, we were able to spatially resolve the distances between individual cell types within TMA tissue cores. We evaluated if the distance between CD8^+^ T cells and RCC cells was different among patients who progressed versus those who did not progress. We demonstrated that patients who progressed to metastatic disease had smaller distances between CD8^+^ T cells and RCC cells (mean distance of 20.72 μm for patients who progressed versus that of 15.92 μm for patients who did not progress, *p* = 0.03; Figure 6C). Additionally, the average CD8^+^ infiltration was higher within a defined radius of 150 pixels around malignant cells for patients that did not progress compared to patients that did progress (Figure 6C). Taking all findings together, the inflammatory response appeared more robust, with a higher interaction between CD8^+^ T cells and ccRCC cells among patients that did not progress to metastatic disease (Figure 6C,D).

## 4. Discussion

This study demonstrates that the composition of immune cells within the tumor microenvironment is spatially heterogeneous but has prognostic capacity in surgically resected, non-metastatic ccRCC. Using multi-region sampling, the degree of CD8^+^ T cell infiltration was prognostic for metastatic progression, and a higher CD8^+^ T cell density was associated with a better prognosis in a discovery cohort and validated in an independent cohort of patients. Furthermore, CD8^+^ T cell infiltration was also associated with early progression to metastasis after surgery. Interestingly, patients who progressed to metastatic disease also had increased percentages of exhausted CD8^+^ T cells. Finally, using spatial analysis, we demonstrated that patients without metastatic progression had CD8^+^ T cells in closer proximity to ccRCC cells. Taken together, these findings demonstrate patterns of immune cell infiltration that are associated with metastatic progression in high-risk RCC, which could be used to identify patients for adjuvant therapy and clinical trials.

Prior studies have evaluated CD8^+^ T cell infiltration as a biomarker but reported conflicting results regarding its prognostic capability [9,16,17,18,26,27]. Early data from Giraldo et al. suggested that increasing CD8^+^ T cell density was associated with worse disease-free and overall survival for RCC patients [17]. This observation is unusual and differs from other solid tumors (e.g., glioma, melanoma, lung adenocarcinoma, and urothelial carcinoma), for which increased CD8^+^ T cell infiltration is associated with a favorable prognosis [28]. Conversely, a later study by Jansen et al. found that CD8^+^ T cell infiltration is associated with better outcomes [18]. There are multiple potential explanations for the conflicting data, which were investigated in this study. First, differences in study design or techniques used for evaluation of immune cell infiltration could significantly confound findings. Second, prior studies used patient cohorts that included both early and advanced-stage tumors that may have different quantities and phenotypes of immune cell infiltration. Finally, immune infiltration is heterogeneous throughout large tumors, as demonstrated in our study, which may skew findings depending on the quantity of immune infiltration in the portion of the tumor that was sampled.

This study was designed in a manner to address some possible differences in study design or techniques that might contribute to conflicting results from prior investigations. We used two independent cohorts and included only those patients who had non-metastatic disease in order to try and reduce the heterogeneity of the immune microenvironment phenotype (e.g., activated versus exhausted) [29]. Additionally, our validation cohort was matched on multiple clinical and pathologic variables known to be associated with progression; thus, differences in outcomes are less likely to be associated with these known confounding covariates. Also, unlike prior studies, our validation cohort had long-term follow-up, which is ideal for studies with non-metastatic patients. Given that over 90% of patients will progress within 10 years of surgery for localized ccRCC, the median follow-up for the validation cohort without progression was 11 years [3]. We focused our analysis on progression-free survival instead of overall survival, which is less likely to be confounded by changes over time in the systemic treatment availability for metastatic RCC (e.g., development-targeted therapy or immune checkpoint therapy). Lastly, we chose to utilize protein expression for the quantification of immune cells, compared to many prior studies that utilized gene expression. Protein expression provides a more direct measurement of the types and states (e.g., exhausted or activated) of immune cells present in the tumor microenvironment rather than inferring the presence of immune cells from gene expression signatures.

The immune microenvironment composition is dynamic during progression from early to advanced tumors, and studies investigating the immune microenvironment as a prognostic biomarker must account for this in their design. Contradictory results regarding the prognostic value of CD8^+^ T cells may result when different-stage tumors are analyzed in aggregate. In the study by Giraldo et al., which suggested that immune infiltration was associated with poor outcomes, over half (54%) of the ccRCC tumors were locally advanced or metastatic (stage III or IV) tumors. In the metastatic setting, other studies have suggested that CD8^+^ T cell infiltration is associated with a worse prognosis, likely attributable to an immune-exhausted state [29,30,31]. Similar to Jansen et al. [18], we found that increasing CD8^+^ T cell infiltration was associated with improved progression-free survival among ccRCC tumors that were non-metastatic. By focusing our analysis for biomarker development on only non-metastatic disease, these data may avoid the confounding influence of different clinical tumor stages on the quantity and type of immune infiltration. Additionally, we demonstrated that increased exhaustion of CD8^+^ T cells, defined by co-expression of PD1^+^ and LAG3^+^, was associated with increased odds of progression. This supports the concept that the immune microenvironment evolves to a more exhausted phenotype in patients who develop advanced disease.

A key objective of this study was to evaluate immune microenvironment heterogeneity within high-risk non-metastatic ccRCC tumors because this subpopulation would benefit from a prognostic biomarker. This study demonstrated that there is substantial variability in immune cell infiltration within large tumors, creating a potential for sampling error in prior studies when only one area is used for biomarker development. The coefficients of variation for all of the immune cells evaluated were high, and the majority of immune cells were located outside the epithelial compartment within the stromal compartment, suggesting the immune cells have limited capacity to penetrate the tumor beyond the invasive margin. Given the high degree of heterogeneity within ccRCC tumors, our study is strengthened in that our validation cohort sampled seven to eight different locations throughout the primary tumor to adequately address issues of tumor heterogeneity and accurately reflect the degree of immune infiltration. While the optimal sampling strategy is not known, a study by the TRACERx Renal Consortium evaluated the number of biopsies that are required to adequately capture the genetic drives of ccRCC [32]. The study suggested that for larger tumors, between four and eight biopsies are needed to capture the majority of genetic driver events [32]. We applied this rationale to our study design by selecting seven to eight different tumor regions for TMA construction in an effort to evaluate tumor heterogeneity. Our findings also have implications for using immune cells as biomarkers. We demonstrate that using only a single or few biopsy locations is unlikely to completely reflect the tumor immune infiltration. Future efforts to evaluate immune infiltration as a prognostic biomarker should be focused on defining an ideal tissue sampling strategy.

We demonstrated that the spatial organization of immune and malignant cells within the primary tumor differs between patients who progress to metastatic disease versus those who do not. Among patients who did not progress, CD8^+^ T cells were more closely associated spatially with tumor cells. It appears that not only the degree of CD8^+^ T cell inflammation but also the organization of these cells around malignant cells is greater among patients who do not progress to metastatic disease. While similar findings have been demonstrated in colorectal cancer, the prognostic value of the spatial organization of immune cells within the non-metastatic ccRCC microenvironment has not been well characterized [33,34].

We showed that CD8^+^ T cell infiltration can help identify patients likely to rapidly progress (within 12 months) after surgery. Both the discovery and validation cohorts demonstrated that higher CD8^+^ T cell infiltration was associated with a reduced risk of rapid progression. Identifying patients at risk of rapid progression is critically important, particularly in light of the newly approved adjuvant immunotherapy, pembrolizumab [8]. With further validation, using CD8^+^ T cell infiltration as a prognostic biomarker could identify patients who may benefit from adjuvant immunotherapy. Currently, adjuvant immunotherapy is approved for a broad, heterogeneous group of patients. Identifying the patients most likely to benefit (i.e., those at highest risk of progression) will reduce the number of patients receiving unnecessary immunotherapy, decreasing both the cost of therapy and exposure to immune-related toxicity [35]. A future study will focus on not only the prognostic value of the immune microenvironment but also its ability to predict response to adjuvant immunotherapy in a non-metastatic setting.

This study has limitations. While we did attempt to address tumor heterogeneity by sampling multiple tumor locations, heterogeneity likely still impacts our results. The techniques used to quantify immune cells have inherent limitations, including the ability of antibodies to bind to cell surface proteins, which may lead to overestimation or underestimation of the number of immune cells present. We attempted to address these issues with multiple quality control measures, but errors are still possible. The findings from this study are primarily associative, and causal mechanisms defining why higher CD8^+^ T cell infiltration is associated with reduced progression cannot be determined from this study alone, requiring further investigation.

## 5. Conclusions

In conclusion, the immune microenvironment in non-metastatic ccRCC is highly heterogeneous. If using components of the immune microenvironment as a potential clinically applicable prognostic biomarker, multiple samples should be obtained, with the optimal strategy to be determined in future studies. Our study demonstrates that CD8^+^ T cells are associated with prognosis. Increased infiltration with non-exhausted CD8^+^ T cells correlated with reduced rates of rapid progression after surgery and improved progression-free survival.

## Figures and Tables

**Table 1:** Clinical and pathologic characteristics of the discovery cohort.

Discovery Cohort	No Progression	Progression	*p* Value
	N = 52	N = 31	
Median age, years (IQR)	58 (54–64)	64 (55–74)	0.03
Gender, no. of females (%)	15 (31)	6 (19)	0.3
ECOG Performance Status, no. (%)			1
0	43 (91)	29 (93)	
1	4 (9)	2 (7)	
Pathologic T-stage			1
T2	2 (4)	1 (3)	
T3–T4	50 (96)	30 (97)	
Median maximum pathologic tumor diameter, cm (IQR)	9.5 (8–11)	9.8 (8–12.5)	0.4
Grade, no. (%)			0.02
1–2	14 (27)	2 (6)	
3–4	38 (73)	29 (94)	
Thrombus	29 (56)	22 (71)	0.2
Died, no. (%)	11 (21)	17 (55)	0.004
Median follow-up, months (IQR)	37 (13–81)	33 (14–77)	0.9

ECOG = Eastern Cooperative Oncology Group, IQR = interquartile range.

**Table 2:** Clinical and pathologic characteristics from the validation cohort.

Validation Cohort	No Progression	Progression	*p* Value
	N = 20	N = 26	
Median age, years (IQR)	61 (51–70)	57 (50–66)	0.3
Gender, no. of females (%)	8 (40)	9 (35)	0.8
ECOG Performance Status, no. (%)			0.6
0	19 (95)	23 (88)	
1	1 (5)	3 (12)	
Median preop NLR, (IQR)	4.4 (2.7–6.1)	2.9 (2.3–4.2)	0.2
Median preop CRP, (IQR)	2 (2–11)	1 (1–3)	0.2
Pathologic T-stage			0.2
T2	10 (50)	8 (31)	
T3–T4	10 (50)	18 (69)	
Median maximum pathologic tumor diameter, cm (IQR)	9 (7.2–9.4)	9.1 (8–13)	0.2
Grade, no. (%)			0.4
1–2	11 (55)	10 (38)	
3–4	9 (45)	16 (62)	
Thrombus	7 (35)	8 (31)	1
Died, no. (%)	5 (25)	14 (54)	0.07
Median follow-up, years (IQR)	11 (8–15)	7 (5–11)	0.08

ECOG = Eastern Cooperative Oncology Group, IQR = interquartile range, NLR = neutrophil lymphocyte ratio, CRP = C-reactive protein.

**Table 3:** Coefficients of variation for immune infiltration among the validation cohort.

Median Coefficient of Variation	No Progression (N = 20)	Progression (N = 26)	*p* Value
CD8 CV	0.86	0.94	0.4
CD4 CV	0.66	0.54	0.2
CD20 CV	0.48	0.51	0.3
CD68 CV	0.79	0.92	0.1

CV = coefficient of variation.

## Data Availability

All data will be made available upon reasonable request to the corresponding author.

## Supplementary Materials

The following supporting information can be downloaded at: https://www.mdpi.com/article/10.3390/cancers16030478/s1, Table S1: PhenoCycler panel used for staining validation tissue microarray.

## References

[1] Siegel R.L., Miller K.D., Wagle N.S., Jemal A. (2023). Cancer statistics, 2023. CA Cancer J. Clin..

[2] Campbell S.C., Clark P.E., Chang S.S., Karam J.A., Souter L., Uzzo R.G. (2021). Renal mass and localized renal cancer: Evaluation, management, and follow-up: Aua guideline: Part I. J. Urol..

[3] Campbell S.C., Uzzo R.G., Karam J.A., Chang S.S., Clark P.E., Souter L. (2021). Renal mass and localized renal cancer: Evaluation, management, and follow-up: Aua guideline: Part II. J. Urol..

[4] Dabestani S., Beisland C., Stewart G.D., Bensalah K., Gudmundsson E., Lam T.B., Gietzmann W., Zakikhani P., Marconi L., Fernandéz-Pello S. (2019). Long-term outcomes of follow-up for initially localised clear cell renal cell carcinoma: Recur database analysis. Eur. Urol. Focus.

[5] Abel E.J., Margulis V., Bauman T.M., Karam J.A., Christensen W.P., Krabbe L.M., Haddad A., Golla V., Wood C.G. (2016). Risk factors for recurrence after surgery in non-metastatic rcc with thrombus: A contemporary multicentre analysis. BJU Int..

[6] Correa A.F., Jegede O., Haas N.B., Flaherty K.T., Pins M.R., Messing E.M., Manola J., Wood C.G., Kane C.J., Jewett M.A.S. (2019). Predicting renal cancer recurrence: Defining limitations of existing prognostic models with prospective trial-based validation. J. Clin. Oncol..

[7] Powles T., Tomczak P., Park S.H., Venugopal B., Ferguson T., Symeonides S.N., Hajek J., Gurney H., Chang Y.H., Lee J.L. (2022). Pembrolizumab versus placebo as post-nephrectomy adjuvant therapy for clear cell renal cell carcinoma (keynote-564): 30-month follow-up analysis of a multicentre, randomised, double-blind, placebo-controlled, phase 3 trial. Lancet Oncol..

[8] Choueiri T.K., Tomczak P., Park S.H., Venugopal B., Ferguson T., Chang Y.-H., Hajek J., Symeonides S.N., Lee J.L., Sarwar N. (2021). Adjuvant pembrolizumab after nephrectomy in renal-cell carcinoma. N. Engl. J. Med..

[9] Şenbabaoğlu Y., Gejman R.S., Winer A.G., Liu M., Allen E.M.V., Velasco G.d., Miao D., Ostrovnaya I., Drill E., Luna A. (2016). Tumor immune microenvironment characterization in clear cell renal cell carcinoma identifies prognostic and immunotherapeutically relevant messenger rna signatures. Genome Biol..

[10] Lasorsa F., Rutigliano M., Milella M., Ferro M., Pandolfo S.D., Crocetto F., Tataru O.S., Autorino R., Battaglia M., Ditonno P. (2023). Cellular and molecular players in the tumor microenvironment of renal cell carcinoma. J. Clin. Med..

[11] Motzer R.J., Tannir N.M., McDermott D.F., Frontera O.A., Melichar B., Choueiri T.K., Plimack E.R., Barthélémy P., Porta C., George S. (2018). Nivolumab plus ipilimumab versus sunitinib in advanced renal-cell carcinoma. N. Engl. J. Med..

[12] Motzer R.J., Penkov K., Haanen J., Rini B., Albiges L., Campbell M.T., Venugopal B., Kollmannsberger C., Negrier S., Uemura M. (2019). Avelumab plus axitinib versus sunitinib for advanced renal-cell carcinoma. N. Engl. J. Med..

[13] Motzer R., Alekseev B., Rha S.Y., Porta C., Eto M., Powles T., Grünwald V., Hutson T.E., Kopyltsov E., Méndez-Vidal M.J. (2021). Lenvatinib plus pembrolizumab or everolimus for advanced renal cell carcinoma. N. Engl. J. Med..

[14] Rini B.I., Plimack E.R., Stus V., Gafanov R., Hawkins R., Nosov D., Pouliot F., Alekseev B., Soulieres D., Melichar B. (2019). Pembrolizumab plus axitinib versus sunitinib for advanced renal-cell carcinoma. N. Engl. J. Med..

[15] Lasorsa F., di Meo N.A., Rutigliano M., Milella M., Ferro M., Pandolfo S.D., Crocetto F., Tataru O.S., Autorino R., Battaglia M. (2023). Immune checkpoint inhibitors in renal cell carcinoma: Molecular basis and rationale for their use in clinical practice. Biomedicines.

[16] Shapiro D.D., Dolan B., Laklouk I.A., Rassi S., Lozar T., Emamekhoo H., Wentland A.L., Lubner M.G., Abel E.J. (2023). Understanding the tumor immune microenvironment in renal cell carcinoma. Cancers.

[17] Giraldo N.A., Becht E., Pagès F., Skliris G., Verkarre V., Vano Y., Mejean A., Saint-Aubert N., Lacroix L., Natario I. (2015). Orchestration and prognostic significance of immune checkpoints in the microenvironment of primary and metastatic renal cell cancer. Clin. Cancer Res..

[18] Jansen C.S., Prokhnevska N., Master V.A., Sanda M.G., Carlisle J.W., Bilen M.A., Cardenas M., Wilkinson S., Lake R., Sowalsky A.G. (2019). An intra-tumoral niche maintains and differentiates stem-like cd8 t cells. Nature.

[19] Bauman T.M., Huang W., Lee M.H., Abel E.J. (2016). Neovascularity as a prognostic marker in renal cell carcinoma. Hum. Pathol..

[20] Black S., Phillips D., Hickey J.W., Kennedy-Darling J., Venkataraaman V.G., Samusik N., Goltsev Y., Schürch C.M., Nolan G.P. (2021). Codex multiplexed tissue imaging with dna-conjugated antibodies. Nat. Protoc..

[21] Zhang W., Li I., Reticker-Flynn N.E., Good Z., Chang S., Samusik N., Saumyaa S., Li Y., Zhou X., Liang R. (2022). Identification of cell types in multiplexed in situ images by combining protein expression and spatial information using celesta. Nat. Methods.

[22] Baitsch L., Baumgaertner P., Devêvre E., Raghav S.K., Legat A., Barba L., Wieckowski S., Bouzourene H., Deplancke B., Romero P. (2011). Exhaustion of tumor-specific cd8+ t cells in metastases from melanoma patients. J. Clin. Investig..

[23] Ahmadzadeh M., Johnson L.A., Heemskerk B., Wunderlich J.R., Dudley M.E., White D.E., Rosenberg S.A. (2009). Tumor antigen–specific cd8 t cells infiltrating the tumor express high levels of pd-1 and are functionally impaired. Blood.

[24] Giles J.R., Globig A.-M., Kaech S.M., Wherry E.J. (2023). Cd8+ t cells in the cancer-immunity cycle. Immunity.

[25] Feng Y., Yang T., Zhu J., Li M., Doyle M., Ozcoban V., Bass G.T., Pizzolla A., Cain L., Weng S. (2023). Spatial analysis with spiat and spasim to characterize and simulate tissue microenvironments. Nat. Commun..

[26] Giraldo N.A., Becht E., Vano Y., Petitprez F., Lacroix L., Validire P., Sanchez-Salas R., Ingels A., Oudard S., Moatti A. (2017). Tumor-infiltrating and peripheral blood t-cell immunophenotypes predict early relapse in localized clear cell renal cell carcinoma. Clin. Cancer Res..

[27] Braun D.A., Hou Y., Bakouny Z., Ficial M., Angelo M.S., Forman J., Ross-Macdonald P., Berger A.C., Jegede O.A., Elagina L. (2020). Interplay of somatic alterations and immune infiltration modulates response to pd-1 blockade in advanced clear cell renal cell carcinoma. Nat. Med..

[28] Varn F.S., Wang Y., Mullins D.W., Fiering S., Cheng C. (2017). Systematic pan-cancer analysis reveals immune cell interactions in the tumor microenvironment. Cancer Res..

[29] Braun D.A., Street K., Burke K.P., Cookmeyer D.L., Denize T., Pedersen C.B., Gohil S.H., Schindler N., Pomerance L., Hirsch L. (2021). Progressive immune dysfunction with advancing disease stage in renal cell carcinoma. Cancer Cell.

[30] Clark D.J., Dhanasekaran S.M., Petralia F., Pan J., Song X., Hu Y., Leprevost F.d.V., Reva B., Lih T.-S.M., Chang H.-Y. (2019). Integrated proteogenomic characterization of clear cell renal cell carcinoma. Cell.

[31] Hakimi A.A., Voss M.H., Kuo F., Sanchez A., Liu M., Nixon B.G., Vuong L., Ostrovnaya I., Chen Y.-B., Reuter V. (2019). Transcriptomic profiling of the tumor microenvironment reveals distinct subgroups of clear cell renal cell cancer—Data from a randomized phase iii trial. Cancer Discov..

[32] Turajlic S., Xu H., Litchfield K., Rowan A., Horswell S., Chambers T., O’Brien T., Lopez J.I., Watkins T.B.K., Nicol D. (2018). Deterministic evolutionary trajectories influence primary tumor growth: Tracerx renal. Cell.

[33] Schürch C.M., Bhate S.S., Barlow G.L., Phillips D.J., Noti L., Zlobec I., Chu P., Black S., Demeter J., McIlwain D.R. (2020). Coordinated cellular neighborhoods orchestrate antitumoral immunity at the colorectal cancer invasive front. Cell.

[34] Bhate S.S., Barlow G.L., Schürch C.M., Nolan G.P. (2021). Tissue schematics map the specialization of immune tissue motifs and their appropriation by tumors. Cell Syst..

[35] Sharma V., Wymer K.M., Joyce D.D., Moriarty J., Khanna A., Borah B.J., Thompson R.H., Costello B.A., Leibovich B.C., Boorjian S.A. (2023). Cost-effectiveness of adjuvant pembrolizumab after nephrectomy for high-risk renal cell carcinoma: Insights for patient selection from a markov model. J. Urol..

