# Supplementary material for: Non-Metastatic Clear Cell Renal Cell Carcinoma Immune Cell Infiltration Heterogeneity and Prognostic Ability in Patients Following Surgery
Source: Cancers (Basel). 2024 Jan 23;16(3):478. doi: 10.3390/cancers16030478 (PMC10854750; doi:10.3390/cancers16030478)
Supplement: Supplementary file 1 [file cancers-16-00478-s001.zip › cancers-2819834-supplementary.pdf]

Supplementary Table S1. PhenoCycler panel used for staining validation tissue microarray

Number	Target	Clone	Barcode	Reporter
1	HLA-A (Core)	EP1395Y	BX004	RX004-AF750
2	CD8	C8/144B	BX026	RX026-ATTO550
3	CD4	EPR6855	BX003	RX003-Cy5
4	CD20	L26	BX007	RX007-AF750
5	CD14	EPR3653	BX037	RX037-ATTO550
6	CD68	KP1	BX015	RX015-Cy5
7	Pan-Cytokeratin	AE1/AE3	BX019	RX019-AF750
8	CD44	156-3C11	BX005	RX005-ATTO550
9	CD11c	118/A5	BX024	RX024-Cy5
10	CD45RO	UCHL1	BX017	RX017-ATTO550
11	CD45	D9M81	BX021	RX021-Cy5
12	LAG3	EPR20261	BX055	RX055-ATTO550
13	CD3e	EP449E	BX045	RX045-Cy5
14	Ki67	B56	BX047	RX047-ATTO550
15	HLA-DR	EPR3692	BX033	RX033-Cy5
16	ICOS	D1K2T	BX054	RX054-AF647
17	CD57	HNK-1	BX049	RX049-AF647
18	TIM3	D5D5R	BX050	RX050-AF647
19	IDO1	V1NC3IDO	BX027	RX027-Cy5
20	PD1	D4W2J	BX046	RX046-AF647
21	PDL1	RM320	BX043	RX043-AF647
